# Conjugated Linoleic Acids Have Anti-Inflammatory Effects in Cultured Endothelial Cells

**DOI:** 10.3390/ijms24010874

**Published:** 2023-01-03

**Authors:** Carina A. Valenzuela, Ella J. Baker, Elizabeth A. Miles, Philip C. Calder

**Affiliations:** 1 School of Human Development and Health, Faculty of Medicine, University of Southampton, Southampton SO16 6YD, UK; 2 Centro de Investigación del Comportamiento Alimentario, Escuela de Nutrición y Dietética, Facultad de Farmacia, Universidad de Valparaíso, Playa Ancha, Valparaíso 2360102, Chile; 3 NIHR Southampton Biomedical Research Centre, University Hospital Southampton NHS Foundation Trust and University of Southampton, Southampton SO16 6YD, UK

**Keywords:** conjugated fatty acids, inflammation, endothelial cells, atherosclerosis

## Abstract

Conjugated linoleic acid (CLA) isomers may have a role in preventing atherosclerosis through the modulation of inflammation, particularly of the endothelium. However, whether low concentrations of CLAs are able to affect basal unstimulated endothelial cell (EC) responses is not clear. The aim of this study was to evaluate the effects of two CLAs (*cis*-9, *trans*-11 (CLA9,11) and *trans*-10, *cis*-12 (CLA10,12)) on the basal inflammatory responses by ECs. EA.hy926 cells (HUVEC lineage) were cultured under standard conditions and exposed to individual CLAs for 48 h. Both CLAs were incorporated into ECs in a dose-dependent manner. CLA9,11 (1 μM) significantly decreased concentrations of MCP-1 (*p* < 0.05), IL-6 (*p* < 0.05), IL-8 (*p* < 0.01) and RANTES (*p* < 0.05) in the culture medium. CLA10,12 (10 μM) decreased the concentrations of MCP-1 (*p* < 0.05) and RANTES (*p* < 0.05) but increased the concentration of IL-6 (*p* < 0.001). At 10 μM both CLAs increased the relative expression of the NFκβ subunit 1 gene (*p* < 0.01 and *p* < 0.05, respectively), while decreasing the relative expression of PPARα (*p* < 0.0001), COX-2 (*p* < 0.0001) and IL-6 (*p* < 0.0001) genes. CLA10,12 increased the relative expression of the gene encoding IκK-β at 10 μM compared with CLA9,11 (*p* < 0.05) and increased the relative expression of the gene encoding IκBα at 1 and 10 μM compared with linoleic acid (both *p* < 0.05). Neither CLA affected the adhesion of monocytes to ECs. These results suggest that low concentrations of both CLA9,11 and CLA10,12 have modest anti-inflammatory effects in ECs. Thus, CLAs may influence endothelial function and the risk of vascular disease. Nevertheless, at these low CLA concentrations some pro-inflammatory genes are upregulated while others are downregulated, suggesting complex effects of CLAs on inflammatory pathways.

## 1. Introduction

The vascular endothelium plays a key role in maintaining vascular homeostasis by regulating vascular tone and permeability. Dysfunction of the endothelium is a pro-inflammatory state characterized by the chronic activation of the endothelium. This increases the risk of atherosclerosis, which is the starting point for cardiovascular disease. Different fatty acids (FAs) have been shown to affect the inflammatory responses of endothelial cells (ECs) [1,2,3,4,5,6], which in turn could have implications in the development or prevention of vascular-related diseases of high prevalence, such as atherosclerosis.

Amongst FAs, conjugated linoleic acids (CLAs) have been well studied in the last few decades [7,8,9,10,11], mostly in research conducted on animals and in vitro models but also due to a high interest in exploring their possible beneficial effects in humans when used as supplements or in enriched foods [12,13,14,15,16]. While most findings in animal models suggest that CLAs have a protective effect against atherosclerosis [11,17,18,19], clinical trials are less consistent depending on the characteristics of the participants, i.e., healthy vs. subjects with risk factors or diagnosis, the CLA doses used and the duration of the intervention. Irrespective of their effects, the mechanisms of action of CLAs are still unclear. The effects of supplemented CLAs reported from the clinical trials in participants with over nutrition, dyslipidaemia or the presence of other risk factors range from a decreased brachial artery flow-mediated dilation (mixed CLAs, 3 g/d for 8 weeks, 80% CLA9,11, 20% CLA10,12) [20], decreased HDL-cholesterol (mixed CLAs with higher dose of 6.8 g/d, for 12 weeks) [21], decreased blood pressure (mixed CLAs, 4.5 g/d for 4 weeks) [22] and increased markers of inflammation, including C-reactive protein (mixed CLAs, 5.5 g/d for 16 weeks) [23], to no effect on inflammatory markers, aortic stiffness, endothelial function or plasma lipids [22,24,25,26]. Evidence from clinical trials with healthy participants is also diverse. Here, CLAs have been reported to decrease plasma triglyceride and VLDL concentrations (mixed CLAs, 5.5 g/d for 5 weeks) [27] and to decrease the expression of intercellular adhesion molecule (ICAM)-1 by monocytes (CLA9,11 or CLA10,12, 2.5 g/day for 8 weeks), although CLA10,12 induced an increased ratio of LDL- to HDL-cholesterol and of total cholesterol to HDL-cholesterol in the same study [28] but had neutral effects in other studies [12,29]. Studies using CLA9,11-enriched dairy products also showed a reduction in inflammation markers in healthy participants [30,31]. Recently, a prospective study in older adults reported that in participants with atherosclerotic cardiovascular disease serum non-esterified CLA was positively associated with carotid intima-media thickness [32]; the CLA isomer measured was not specified but is likely to be CLA9,11.

There is some evidence showing the CLAs have anti-inflammatory effects in endothelial cells (ECs). This evidence mainly comes from in vitro studies that use ECs stimulated with pro-inflammatory agents, such as lipopolysaccharide (LPS), tumour necrosis factor (TNF)-α, interleukin (IL)-1β or oxidized LDL [33,34,35]. We previously showed that CLA9,11 and CLA10,12 could modulate the response of cultured ECs to TNF-α, sometimes having opposite effects to one another [36]. We now aim to investigate whether those CLAs have effects in their own right (i.e., in the absence of a pro-inflammatory agent) on inflammatory processes in ECs.

## 2. Results

### 2.1. Viability of EA.hy926 Cells Incubated with FAs

**Figure 1:**
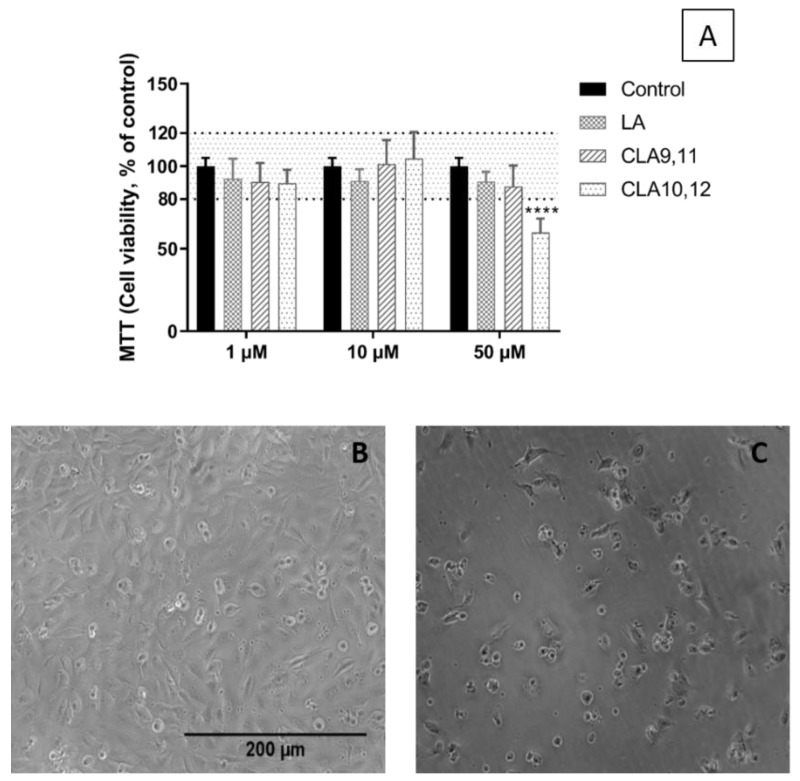
(**A**) Viability of EA.hy926 cells after preincubation for 48 h with medium a containing 0.1% of ethanol (Control) or different concentrations (1, 10 or 50 µM) of linoleic acid (LA), *cis*-9, *trans*-11 linoleic acid (CLA9,11) or *trans*-10, *cis*-12 linoleic acid (CLA10,12) followed by incubation with a medium without fatty acids for 24 h. Bars are mean ± SD of 9 samples from 3 experiments. Data were analysed using one-way ANOVA with Tukey’s post hoc test. **** *p* < 0.0001 vs. Control. (**B**,**C**) Microscope images of EA.hy926 cells incubated with medium containing 0.1% ethanol (**B**) or containing *trans*-10, *cis*-12 linoleic acid at 50 µM (**C**).

The 3-(4,5-dimethylthiazol-2-yl)-2,5-diphenyltetrazolium bromide (MTT) assay was used to test the viability of ECs cultured with CLA9,11 or CLA10,12 or linoleic acid (LA) as a comparator. None of the three FAs affected the viability of EA.hy926 cells when tested at concentrations of 1 and 10 µM (Figure 1). However, CLA10,12 at a concentration of 50 µM decreased the cell viability significantly (*p* < 0.0001). Therefore, in further experiments, FAs were used only at concentrations of 1 and 10 µM.

### 2.2. FA Incorporation into EA.hy926 Cells

As reported previously [36], each of the studied FAs was incorporated into EA.hy926 cells in a concentration-dependent manner as their concentration in the culture medium increased from 1 to 10 µM.

### 2.3. Effects of FAs on the Concentrations of Inflammatory Mediators in the Medium of Cultured ECs

**Figure 2:**
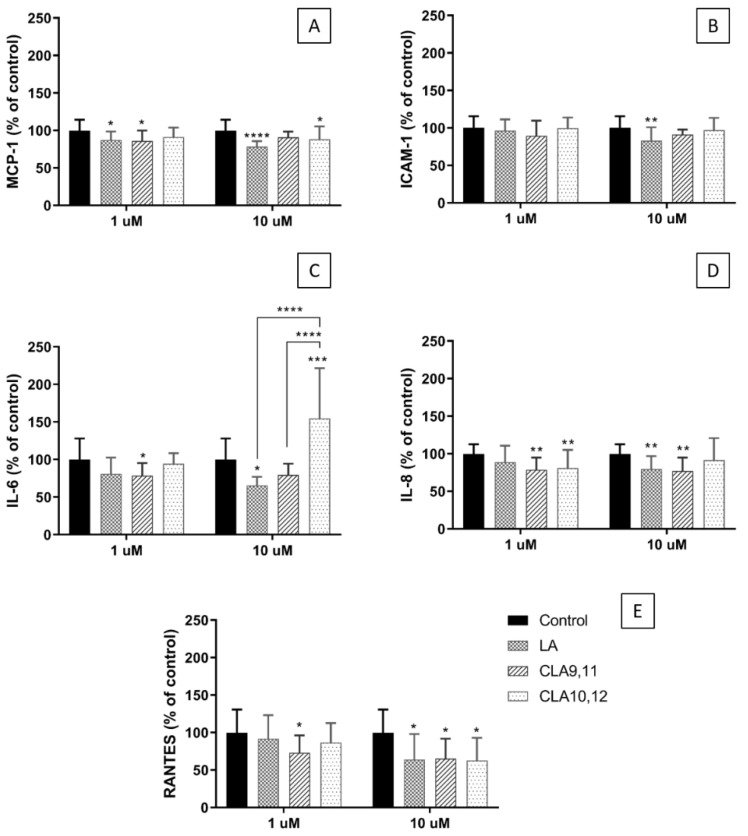
Concentrations (% of control) of MCP-1 (**A**), ICAM-1 (**B**), IL-6 (**C**), IL-8 (**D**) and RANTES (**E**) in the medium of EA.hy926 cells incubated for 48 h with medium containing 0.1% of ethanol (Control) or fatty acid at 1 or 10 µM, followed by incubation with a medium without fatty acids for 24 h. Bars are mean ± SD of 9 samples from 3 experiments. Data were analysed using the one-way ANOVA with Tukey’s post hoc test. * *p* < 0.05; ** *p* < 0.01; *** *p* < 0.001; **** *p* < 0.0001; where asterisks are shown immediately above a bar they refer to the difference from the control and where asterisks are shown above a horizontal line they refer to the differences between the two groups indicated by that line. LA, linoleic acid; CLA9,11, *cis*-9, *trans*-11 linoleic acid; CLA10,12, *trans*-10, *cis*-12 linoleic acid.

Incubation of EA.hy926 cells with CLA9,11 at 1 µM decreased the concentration of monocyte chemoattractant protein (MCP)-1 when compared to the control cultures (no added FA). CLA10,12 also decreased the concentration of MCP-1 when used at 10 µM (*p* < 0.05). The all-*cis* isomer, LA, decreased the concentration of MCP-1 when used at either 1 or 10 µM (Figure 2A). Neither CLA isomer affected the supernatant concentration of intercellular adhesion molecule (ICAM)-1 (Figure 2B). However, incubation with LA at a concentration of 10 µM resulted in a significant decrease (*p* < 0.0001) in the concentration of ICAM-1 in the medium (Figure 2B). Incubation with CLA9,11 significantly decreased interleukin (IL)-6 concentration when used at 1 µM (*p* < 0.05) (Figure 2C). In contrast, CLA10,12 at 10 µM increased the concentration of IL-6 (*p* < 0.001), behaving differently from LA and CLA9,11 (Figure 2C). Both CLAs at a concentration of 1 µM resulted in a decreased IL-8 concentration in the medium (*p* < 0.01) (Figure 2D). CLA9,11 and LA also resulted in a decreased IL-8 concentration when used at 10 µM (*p* < 0.01) (Figure 2D). Incubation with CLA9,11 at 1 µM resulted in a decreased regulated upon activation, normal T cell expression and presumably secreted (RANTES) concentration (*p* < 0.05) (Figure 2E). When used at 10 µM both CLAs and LA resulted in decreased RANTES concentration (*p* < 0.05) (Figure 2E).

### 2.4. Effects of FAs on the Expression of Inflammation-Related Genes

**Figure 3:**
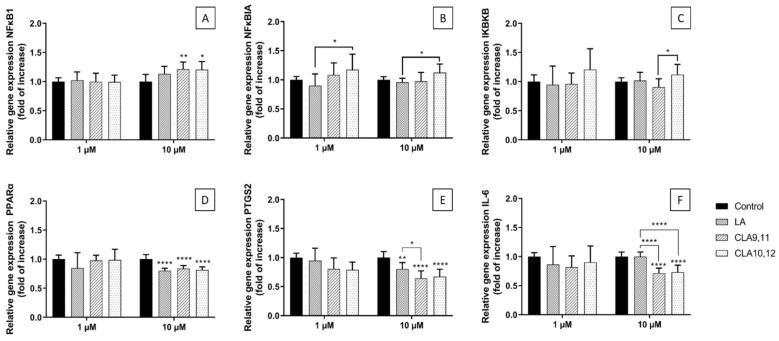
Expression of *NFκB1* (**A**), *NFκBIA* (for IκBα, (**B**)), IκBKB (for IκK-β, (**C**)), *PPPAR-α* (**D**), *PTGS2* (for COX-2, (**E**)) and *IL-6* (**F**) genes in EA.hy926 cells preincubated for 48 h with 1 or 10 µM of fatty acid in a medium containing 0.1% of ethanol (Control) followed by incubation with a medium without fatty acids for 6 h. Cq values were normalized by the geometric mean of reference targets (YWHAZ and RPL13A genes). Bars are mean ± SD of 9 samples from 3 experiments. Data were analysed using the one-way ANOVA with Tukey’s post hoc test. * *p* < 0.05, ** *p* < 0.01, **** *p* < 0.0001; where asterisks are shown immediately above a bar they refer to the difference from the control and where asterisks are shown above a horizontal line they refer to the differences between the two groups indicated by that line. LA, linoleic acid; CLA9,11, *cis*-9, *trans*-11 linoleic acid; CLA10,12, *trans*-10, *cis*-12 linoleic acid.

Incubating ECs with either CLA9,11 or CLA10,12 at a concentration of 10 µM resulted in a significant increase in the relative expression of the nuclear factor kappa-light-chain-enhancer of activated cells 1 (NFκB1) gene (*NFκB1*) when compared to the control (*p* < 0.01 and *p* < 0.05, respectively) (Figure 3A). LA was without effect. Incubation with CLA10,12 at a concentration of 1 or 10 μM resulted in a higher expression of *NFkBIA*, the gene encoding an inhibitory subunit of NFκB (IκBα), than that observed when cells were incubated with LA (both *p* < 0.05) (Figure 3B). CLA10,12 at a concentration of 10 μM resulted in a higher expression of *IκBKB*, the gene encoding one of the kinase enzymes that phosphorylates the inhibitory subunit of NFκB, than that observed with CLA9,11 (*p* < 0.05) (Figure 3C). This effect was also seen with CLA10,12 at 1 μM, although it was not significant (Figure 3C).

When used at 10 µM, both CLAs and LA decreased the relative expression of the PPAR-α gene when compared to the control (*p* < 0.0001) (Figure 3D). Similarly, incubation of ECs with either of the CLAs or LA at 10 µM decreased the expression of the gene encoding COX-2 (*PTGS2*) when compared to the control (*p* < 0.0001 for both CLAs; *p* < 0.01 for LA) (Figure 3E). When the CLAs were used at a concentration of 1 µM there was a trend for the decreased expression of *PTGS2* (*p* = 0.052 and 0.08, for CLA9,11 and CLA 10,12 respectively). Both CLAs at 10 µM decreased the expression of the gene encoding IL-6 when compared to the control (*p* < 0.0001) acting significantly differently from LA (*p* < 0.0001) (Figure 3F). LA itself did not affect the IL-6 gene expression (Figure 3F).

### 2.5. Effects of FAs on THP-1 Adhesion to EA.hy926 Cells

**Figure 4:**
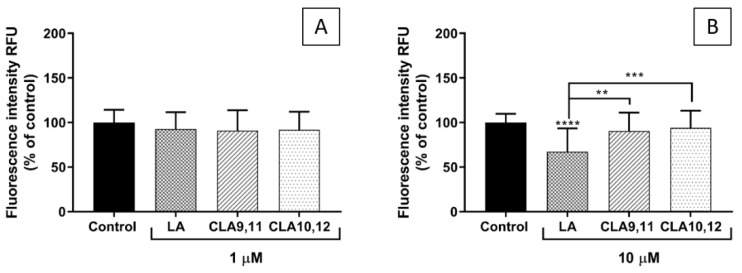
Adhesion of THP-1 cells (% of control) to EA.hy926 cells incubated for 48 h with a medium containing 0.1% of ethanol (Control) or different concentrations (1 µM (**A**), 10 µM (**B**)) of fatty acid, followed by incubation with a medium without fatty acids for 6 h and then 1 h co-incubation with THP-1 cells. Bars are mean ± SD of 9 samples from 3 experiments. Data were analysed using the one-way ANOVA with Tukey post hoc test. ** *p* < 0.01; *** *p* < 0.001; **** *p* < 0.0001; where asterisks are shown immediately above a bar they refer to the difference from the control and where asterisks are shown above a horizontal line they refer to the differences between the two groups indicated by that line. LA, linoleic acid; CLA9,11, *cis*-9, *trans*-11 linoleic acid; CLA10,12, *trans*-10, *cis*-12 linoleic acid.

Pre-incubation of EA.hy926 cells with any of the three FAs at 1 µM did not significantly affect the subsequent adhesion of THP-1 cells (Figure 4A). In contrast, pre-incubation with LA at a concentration of 10 µM resulted in a significant decrease in the adhesion of THP-1 cells when compared with the control and also with each of the CLAs (Figure 4B).

**Figure 5:**
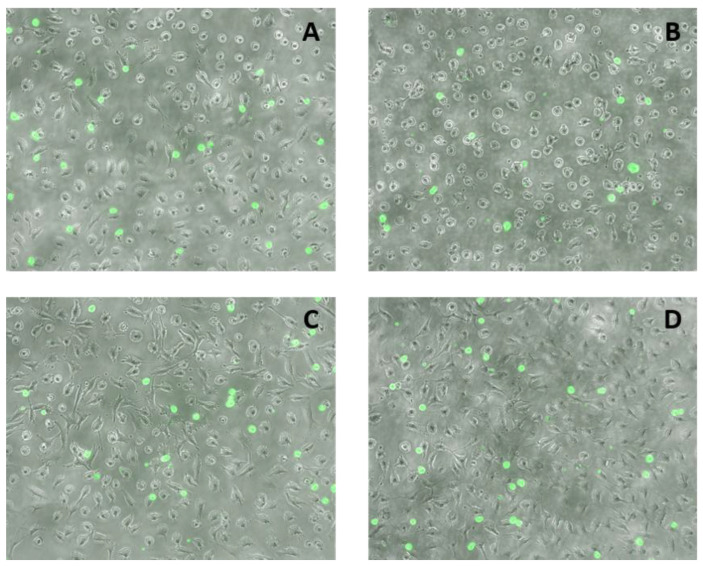
Images of THP-1 cell adhesion to EA.hy926 cells. Adhesion of THP-1 cells to EA.hy926 cells without pre-incubation with fatty acid (control (**A**)) or with 48 h prior exposure to 10 µM linoleic acid (**B**), *cis*-9, *trans*-11 linoleic acid (**C**), *trans*-10, *cis*-12 linoleic acid (**D**), followed by incubation with a medium without fatty acids for 6 h and then 1 h co-incubation with calcein-labelled THP-1 cells. Attached THP-1 cells were visualised by fluorescence microscopy (Nikon Elipse Ti) at a magnification of 100× under transmitted light.

The fluorescence microscopy images support the quantitative results, showing a lower number of THP-1 monocytes (green spots) adhering when ECs were pre-incubated with LA at a concentration of 10 μM (Figure 5B).

## 3. Discussion

We have previously shown the differential effects of CLA9,11 and CLA10,12 at low concentrations (1 and 10 μM) on the inflammatory responses of ECs stimulated with TNF-α [36]. In the current study, the CLAs were used at the same concentrations as previously, but without the presence of an inflammatory stimulus to test if they have a direct effect on the inflammatory response of ECs. In this model, EA.hy926 cells were incubated for 48 h with CLA9,11 or CLA10, 12 or LA and then incubated for 6 or 24 h without fatty acids prior to measuring gene expression (6 h cultures), adhesion of monocytes (6 h cultures) or inflammatory mediator concentrations in the culture medium (24 h cultures). This approach was taken in order to investigate the effects of EC enrichment with the FAs on subsequent inflammatory responses. CLA9,11 had some anti-inflammatory effects in non-stimulated ECs, decreasing the concentrations of MCP-1, RANTES, IL-8 and IL-6 in the culture medium. CLA10,12 also decreased MCP-1 and RANTES concentrations but increased the concentration of IL-6 when used at 10 µM. In accordance with these generally anti-inflammatory effects, both CLAs decreased COX-2 and IL-6 gene expression. The effects of CLA9,11 on IL-6 gene expression and the IL-6 concentration in the culture medium, both of which were decreased, may be related. However, CLA10,12 decreased IL-6 gene expression but increased IL-6 concentration in the culture medium. It is not clear why these findings are contradictory. Both CLAs decreased PPAR-α gene expression. PPAR-α has anti-inflammatory actions [37] and therefore the effect of CLAs in decreasing PPAR-α gene expression is in contrast to the decreases in the inflammatory mediator concentrations seen in the culture medium. Since both CLAs decreased the concentrations of some of the pro-inflammatory mediators measured in the culture medium, the higher gene expression of NFκB1 seen after incubation with both CLAs may not have translated into the higher expression of its protein or its activity, neither of which were measured here. The complexity of the effects on this system is apparent because CLA10,12 increased the expression of the genes encoding both a kinase that activates the NFκB system and encoding one of the inhibitory subunits of NFκB. Therefore, the overall effect on the NFκB system is difficult to predict based upon these gene expression measurements. Both CLA isomers had neutral effects on THP-1 adhesion to ECs, when compared to the control at both of the concentrations used. It is worth noting that the comparator LA had some effects at the concentrations used and these were generally anti-inflammatory, including decreasing the concentrations of MCP-1, ICAM-1, IL-6, IL-8 and RANTES, decreasing PTGS2 (COX-2) gene expression and decreasing THP-1 adhesion to ECs when used at 10 μM, although, again, LA decreased the PPAR-α gene expression.

The results suggest that both CLAs can have anti-inflammatory effects in their own right (i.e., in the absence of an inflammatory stimulus) although CLA10,12 did induce an increase in IL-6 concentration, which may be considered as a pro-inflammatory effect. However, IL-6 does have pleiotropic actions [38] and low concentrations of IL-6 have been shown to have anti-inflammatory effects.

Other authors have also reported neutral or beneficial effects of CLAs in the modulation of inflammation. Tricon et al. reported that supplementation with CLA9,11 or CLA10,12 in 49 healthy men had no effect on C-reactive protein levels, body composition or insulin concentration, but the higher dose of both CLAs (2.38 and 2.52 g/d, respectively) decreased the number of monocytes expressing ICAM-1. In the same study, CLA10,12 increased the LDL- to HDL-cholesterol ratio, whereas CLA9,11 decreased it [13,28]. In another study, 29 healthy adult volunteers underwent a CLA depletion followed by an 8 week period consuming 20 g of CLA9,11 enriched butter daily (1020 ± 167 mg CLA/day); when compared to the end of the depletion phase, the CLA repletion resulted in decreased NFκB protein in blood mononuclear cells, decreased serum levels of TNF-α, IL-2 and IL-8 and increased levels of the anti-inflammatory cytokine IL-10 [31]. Raff et al. found no effect of an enriched butter with CLA9,11 and CLA10,12 (5.5 g/d, mixture of 39.4% CLA9,11 and 38.5% CLA10,12) for 5 weeks on plasma total, LDL- and HDL-cholesterol or triglycerides, on inflammatory and haemostatic risk markers, or on fasting insulin and glucose concentrations in healthy young men, although they showed increases in lipid peroxidation [29]. A recently published meta-analysis of 18 randomized controlled trials reported that CLA consumption did not change blood pressure or vascular cell adhesion molecule (VCAM) concentrations, although it may decrease ICAM-1 concentration, a marker of endothelial activation [39].

There are not many pre-clinical studies investigating the effects of CLAs on endothelial function or inflammation in models using healthy animals or unstimulated ECs or other cell lines, which makes it difficult to compare our findings to the literature. Soto-Vaca et al. showed that human coronary artery smooth muscle cells exposed to very high concentrations of CLA or LA (200 µM for 20 h) produced lower supernatant levels of IL-6 when compared to control cultures, while LA also decreased the level of MCP-1 [40]. It is important to note that in that study the authors considered the FA treatments as an inflammatory stimulus, although their results do not support this assumption. Stachowska et al. showed that the incubation of monocytes from healthy donors with CLA9,11 and CLA10,12 at a concentration of 100 µM for 7 days decreased the expression of the integrins VLA-4 and Mac-1 [34]. Both CLA9,11 or CLA10,12 at a concentration of 100 μM resulted in the decreased expression of VCAM-1 on the surface of ECs but only CLA9,11 decreased the cell surface ICAM-1 expression when compared to a control [34]. Additionally, both CLA isomers showed a strong tendency to reduce the binding of monocytes to HUVECs [34].

Interestingly, LA showed some anti-inflammatory effects in the current study, inducing a decrease in the concentration of several inflammatory mediators measured in the culture supernatant, downregulating the expression of the gene encoding COX-2 and also decreasing monocyte adhesion to ECs when used at 10 µM. It is important to consider that the LA concentrations used were quite low, in comparison to the concentrations present in human blood. For example, the plasma concentration of LA reported in young healthy Canadian adults was 2233.8 ± 622.6 μM [41].

In summary, the results of the current study show that both CLA9,11 and CLA10,12 can have an effect in their own right on inflammatory responses in ECs when used at a low concentration. They showed modest anti-inflammatory effects, which may influence endothelial function and the risk of developing vascular disease. Despite the generally anti-inflammatory effects of CLAs seen on the concentrations of inflammatory mediators in the culture medium, at the low CLA concentrations used, some pro-inflammatory genes were upregulated while others were downregulated. This suggests a complex effect of CLAs on the inflammatory pathways in unstimulated ECs.

## 4. Materials and Methods

### 4.1. Endothelial Cells

EA.hy926 cells, ECs of the HUVEC lineage, were purchased from American Type Culture Collection, LGC Standards, Teddington, UK. They were cultured in high glucose Dulbecco’s Modified Eagle Medium (DMEM) supplemented with 10% foetal bovine serum, 1% L-glutamine-penicillin-streptomycin solution, 1% HAT (100 µM hypoxanthine, 0.4 µM aminopterin and 16 µM thymidine). Medium and medium supplements were all purchased from Sigma-Aldrich (Gillingham, UK). The EA.hy926 cell cultures were maintained at 37 °C in humidified 95% air and 5% CO_2_. Before their use in the experiments ECs were grown in T-175 flasks (Corning, Corning, NY, USA) until confluent.

### 4.2. Fatty Acid Treatment

*Cis*-9, *trans*-11 linoleic acid (CLA9,11), *trans*-10, *cis*-12 linoleic acid (CLA10,12), and linoleic acid (LA) were purchased from Cayman Chemicals, Cambridge, UK. They were prepared as stock solutions (1, 10 and 50 mM) in 100% ethanol. Prior to each experiment, the stock solutions were diluted in warm complete culture medium to give a final FA concentration of 1, 10 and 50 μM; the final ethanol concentration was 0.1%. The corresponding control was a 0.1% ethanol solution diluted in complete medium. For the experiments, the EA.hy926 cells were seeded in 96-well plates (for cell viability, inflammatory mediator and adhesion assays), 6-well plates (for gene expression assessment) and T25 flasks (for FA analysis), cultured in complete medium and exposed to different FAs for 48 h.

### 4.3. MTT Assay for Cell Viability

Cell viability was assessed using the 3-(4,5-dimethylthiazol-2-yl)-2,5-diphenyltetrazolium bromide (MTT) assay, which measures cellular mitochondrial activity (i.e., this is an assay for metabolic integrity). After EC culture, supernatants were removed and replaced with DMEM containing 0.05 mg/mL MTT (Sigma-Aldrich, Gillingham, UK) (100 µL/well) and samples were incubated at 37 °C for 4 h. Supernatants (75 µL) were removed and 75 µL of dimethylsulphoxide (Sigma-Aldrich, Gillingham, UK) was added. Absorbance was measured at 540 nm on a plate reader. The effects of FAs on the cell viability were normalized to control (i.e., no FA, 0.1% ethanol) cultures set to 100%.

### 4.4. Fatty Acid Composition Measurement by Gas Chromatography

The FA concentrations in the culture medium and the FA composition of EA.hy926 cells after culture with the FAs of interest were determined as reported elsewhere [36].

### 4.5. Measurement of Inflammatory Mediators in Cell Culture Supernatants Using Multiplex Magnetic ELISA

Cell culture supernatants were assayed using the Human Magnetic Luminex Screening Assay ELISA (R&D Systems, Minneapolis, MN, USA) to measure the concentrations of monocyte chemoattractant protein (MCP)-1, interleukin (IL)-6, IL-8, regulated upon activation, normal T cell expression and presumably secreted (RANTES) and intercellular adhesion molecule (ICAM)-1. The EA.hy926 cells were incubated with the FAs in 96-well flat-bottomed plates (Corning Corning, NY, USA) (1 × 10^4^ cells/100 µL per well) for 48 h and then without FAs for 24 h. Supernatants were collected and stored at −80 °C until analysis. Assays were conducted in accordance with the manufacturer’s instructions. Plates were analysed on a calibrated Bio-Plex 200 analyser using Bio-Plex software (version 6.1, Bio-Rad Laboratories Inc., Berkeley, CA, USA). Lower limits of detection (pg/mL) were IL-6, 1.7; IL-8, 1.8; MCP-1, 9.9; RANTES, 1.8; ICAM-1, 87.9. Due to the differences in the ranges of fluorescence values among the experiments the results are presented as % of control.

### 4.6. RNA Isolation, cDNA Synthesis, and Real-Time PCR

Changes in the relative gene expression were analysed by RT-PCR. The EA.hy926 cells were cultured in 6-well plates (Corning, Corning, NY, USA) (cell density of 6 × 10^5^ cells/mL) with FAs for 48 h and then without FAs for 6 h. Taqman Gene Expression Primers (Thermo Fisher Scientific, Waltham, MA, USA) were used to determine the expression of *NFκB1* (Hs00765730_m1), *NFκBIA* (Hs00355671_g1), *IκBKB* (Hs00233287_m1), *PPARα* (Hs00947536_m1), *PTGS2* (Hs00153133_m1) and *IL-6* (HS00985639_m1). Total RNA was extracted from the cells using the ReliaPrep RNA cell Miniprep System (Promega, Southampton, UK). RNA quantity and quality were analysed by NanoDrop. RNA integrity was assessed as RIN score using an Agilent Bioanalyzer (RNA Total Eukaryote 2100 Nano). cDNA was synthesised from total RNA using GoScript Reverse Transcriptase (Promega, Southampton, UK). Housekeeping reference genes were determined using a geNorm Kit (Primerdesign, Camberley, UK): *YWHAZ* (Hs01122445_g1) and *RPL13A* (Hs04194366_g1) were used as housekeeping genes to determine quantitative gene expression.

### 4.7. Adhesion of THP-1 Monocytes to ECs

The adhesion of THP-1 monocytes to the EA.hy926 cells was determined using the Vybrant Cell Adhesion Assay Kit (ThermoFisher Scientific, Waltham, MA, USA). The EA.hy926 cells were seeded in 96-well flat-bottomed plates (Corning, Corning, NY, USA) (cell density of 2 × 10^5^ cells/mL, 1 × 10^5^ cells per well). After incubation with FAs for 48 h, ECs were incubated with DMEM for 6 h. Calcein-labelled THP-1 cells (5 × 10^4^ cells in 100 µL) were then incubated with the EA.hy926 cells for 1 h at 37 °C. The co-cultures were carried out in high glucose DMEM supplemented with 10% fetal bovine serum and 1% L-glutamine-penicillin-streptomycin solution. Non-adherent THP-1 cells were removed by gentle washing and 100 µL phosphate-buffered saline (PBS) was added to each well and the co-cultures were read on the Glomax Discover System (Promega, Southampton, UK). THP-1 monocyte adhesion was measured as a percentage of control (ECs incubated with DMEM, and then with calcein-labelled THP-1 cells). Images of fluorescence-labelled THP-1 monocytes bound to the EA.hy926 cells were taken with a Nikon Elipse Ti using NIS elements software (version 4.30, Nikon Instruments, Amsterdam, The Netherlands).

### 4.8. Data Analysis

Data are presented as mean ± SD and were analysed by the one-way ANOVA followed by Tukey’s post hoc test for pairwise differences. Analyses were performed using GraphPad Prism 6.0 (Graph Pad, San Diego, CA, USA). Differences were considered significant when *p* < 0.05.

## Data Availability

Data can be made available by contacting the corresponding author.
